# Potential for patient-physician language discordance in Ontario

**DOI:** 10.1186/1472-6963-13-535

**Published:** 2013-12-28

**Authors:** Jennifer Sears, Kamran Khan, Chris I Ardern, Hala Tamim

**Affiliations:** 1 School of Kinesiology and Health Science, York University, Toronto, Canada; 2 Department of Medicine, Division of Infectious Diseases, University of Toronto, Toronto, Canada; 3 Keenan Research Centre, Li Ka Shing Knowledge Institute, St. Michael’s Hospital, Toronto, Canada; 4 York University, 4700 Keele Street, Toronto, Ontario M3J 1P3, Canada

**Keywords:** Primary care medicine, Immigrant health, Access to care, Language discordance

## Abstract

**Background:**

Patient-Physician language discordance occurs when the patient and physician lack proficiency in the same language(s). Previous literature suggests language discordant clinical encounters compromise patient quality of care and health outcomes. The objective of this study was to quantify and visualize the linguistic and spatial mismatch between Ontario’s population not proficient in English or French but proficient in one of the top five non-official languages and the physicians who are proficient in the same non-official language.

**Methods:**

Using data from the 2006 Canadian census and the 2006 Canadian Medical Directory, we determined the number of non-English/non-French (NENF) speaking individuals and the number of Ontario physicians proficient in the top five non-official languages in each census division (CD) of Ontario. For each non-official language, we produced bi-variate choropleth maps of Ontario, broken down into the 49 CDs, to determine which CDs had the highest risk of language discordant clinical encounters.

**Results:**

According to the 2006 Canadian census, the top five non-official languages spoken by Ontario’s NENF population were: Chinese, Italian, Punjabi, Portuguese and Spanish. For each of the top five non-official languages, there were at least 5 census divisions with a NENF population speaking a non-official language without any primary care physicians proficient in that non-official language. The size of NENF populations within these CDs ranged from 10 individuals to 1,470 individuals.

**Conclusions:**

Understanding the linguistic capabilities of Ontario’s immigrant population & the linguistic capabilities of Ontario’s primary care physicians is essential to ensure equal access and quality of healthcare. As immigration continues to increase, we may find that the linguistic needs of Ontario’s immigrant population diverge from the linguistic capabilities of Ontario’s primary care physicians. Further research on the language discordance in Ontario is needed in order to reduce the risk of language discordant clinical encounters and the negative health outcomes associated with these encounters.

## Background

Communication is fundamental to health care access and delivery. The quality of communication between the physician and the patient affects the diagnosis, treatment and recovery of patients [1]. It is especially important that primary care physicians are able to effectively communicate with patients as primary care physician’s are often the first point of access in the health care system. Despite these findings, there is still considerable evidence that physicians’ communication skills can be sub-optimal. Patients often report a desire for increased participation and information sharing [2,3]. Facilitating effective communication becomes even more challenging when language barriers between the physician and patient are introduced.

Language discordance occurs when the patient and the health care professional lack proficiency in the same language(s). There is a growing body of literature that suggests language discordant clinical encounters can seriously compromise patient quality of care and health outcomes [4-9]. Studies have shown that patients with limited proficiency in the physicians’ language(s) were more likely to have longer emergency room stays and in-hospital admissions [6], undergo more diagnostic tests [10], and were less likely to be referred for follow-up appointments [8]. In addition to negative health outcomes, language barriers are associated with increased costs to the health care system; it is associated with increased diagnostic testing and increases in length of hospital stay [11].

In Canada, foreign-born individuals comprise one fifth of the total population [12]. Between 2001 and 2006 Canada’s foreign-born population increased by 13.6%; four times higher than the growth-rate of the Canadian born population during the same period [13]. Given the increase in immigration rates, it might be reasonable to assume that there will be more patients requiring health care services in languages other than English or French. The province of Ontario receives the highest number of new immigrants annually, compared to the rest of Canada. Within the immigrant population, more than 70% report a mother-tongue different from English or French, and according to the 2006 Canadian census, 2.5% of Ontario’s population could not conduct a conversation in either English or French. Previous studies, performed in Ontario, which looked at the health outcomes of patients who lacked proficiency in English found that these patients had longer hospital stays [6], were less likely to use preventative services [9] and had a higher risk of death from Tuberculosis [4] when compared to English speaking patients.

Based on the published literature on patient-physician language discordance, it appears that the ideal circumstance for optimal communication in the health care setting is for both physicians (especially primary care physicians) and patients to be proficient in the same language. We believe that greater awareness of the linguistic proficiency and the spatial distribution of Ontario’s primary care physicians and Ontario’s immigrant population who lack proficiency in English and/or French will help to identify where linguistic gaps are greatest. The objective of this study is to quantify and visualize the linguistic and spatial mismatch between Ontario’s non-English and/or non-French speaking (NENF) population, who are proficient in Ontario’s top five non-official languages, and Ontario’s primary care physicians who are proficient in the same non-official languages.

## Methods

This study involved the analysis and integration of data obtained from two publically available data sources; the 2006 Canadian census and the 2006 Canadian Medical Directory Physician Database. The Canadian census is a nationwide mandatory survey, conducted every five years, that enumerates the entire population of Canada. One person from each household is responsible for completing the census questions for every member in that household. Data from the 2006 Canadian Census are openly available from the Statistics Canada website. The 2006 Canadian Medical Directory (CMD) Physician Database is a national file published annually that contains information on approximately 60,000 physicians practicing medicine across Canada. Over 98% of physicians practicing in Canada are included in the database. These files are available, for purchase, to the general public from Scotts Medical Directory (previously known as the Canadian Medical Directory). The file used in this study was purchased by a co-author of this study (K. Khan).

First, openly available data on mother tongue (by knowledge of official language) was drawn from the Statistics Canada website [13]. The questions from the 2006 Canadian census that were used to create these tabulations were: 1) “Can this person speak English or French well enough to conduct a conversation?” A) English only; B) French only; C) Both English and French; D) Neither English or French and 2) “What is the language that this person first learned at home in childhood and still understands?” A) English; B) French; or C) Other. The tabulations were downloaded for each of Ontario’s 49 census divisions. Census divisions are the second-level of geographical analysis (one-step below provinces and territories) defined by Statistics Canada. For this analysis, individuals who answered neither English nor French to the first question were defined as the non-English/non-French speaking (NENF) population. The top five ‘Other’ languages indicated in question two by the NENF population were defined as the top five non-official languages.

To identify the primary care physicians who spoke one or more of the top five non-official languages we used the ‘Other Language’ variable in the 2006 Canadian Medical Directory, a string variable that allows physicians to input up to three languages. We determined the number of primary care physicians proficient in one or more of the top five non-official languages in each of the 49 census divisions of Ontario. We joined the two datasets using the census division variable, and subsequently calculated a ratio of primary care physicians to number of NENF individuals for each of the top five non-official languages in each census division. Bivariate choropleth maps of Ontario, broken down into the 49 census divisions, were then used to display the number of NENF individuals who speak language ‘X’ by the number of primary care physicians speaking language ‘X’. Bivariate choropleth maps compare the distribution of two variables across the same geography. Choropleth maps use varying different shades of colours within defined geographic boundaries to represent the different quantities of that variable. In our analysis, the census divisions shaded pink had primary care physicians who are capable of speaking language ‘X’ but did not have a NENF population speaking language ‘X’. Darker shades of pink represent a higher number of primary care physicians. Conversely, the census divisions shaded blue had a NENF population speaking language ‘X’ but did not have primary care physicians capable of speaking language ‘X’. Darker shades of blue represent a larger NENF population. The census divisions shaded white did not have physicians or a NENF population speaking language ‘X’ and census divisions shaded in the darkest shade of purple had the highest number of physicians speaking language ‘X’ and the largest NENF population speaking language ‘X’. Census divisions shaded in pink or blue represent discordant census divisions where as census divisions shaded in purple represent concordant census divisions. All maps were created using ArcMap GIS software. Both population and physician class breaks were determined by using the Jenk’s Natural Breaks [14] statistical method built into ArcMap.

## Results

**Figure 1:**
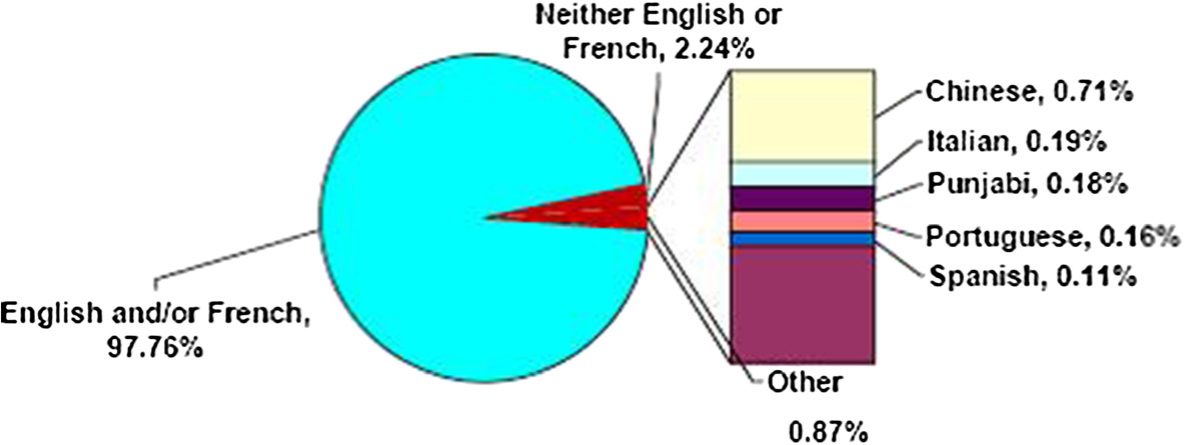
The distribution of languages spoken by the Ontario population in 2006.

According to the tabulations from the 2006 Canadian census, 265,335 individuals living in Ontario could not conduct a conversation in English or French and specified a non-official mother tongue. These individuals represented 2.24% of the population of Ontario (approximately 12M people). The distribution of languages spoken by Ontario’s population is displayed in Figure 1. There were over 100 languages indicated by the NENF population, however the top five non-official languages were found to be: Chinese (37,070 individuals), Italian (22,900 individuals), Punjabi (21,250 individuals), Portuguese (19,360 individuals) and Spanish (13,454 individuals). The distribution of the top ten non-official languages spoken in Ontario in 2006 is displayed in Table 1; we grouped the rest of the languages indicated as “other languages” for simplicity. In 2006, there were 10, 257 primary care physicians practicing in Ontario. Approximately 18% of the primary care physicians indicated they were proficient in one or more languages other than English or French; only 3.67% indicated they were proficient in Chinese, Italian, Punjabi, Portuguese and/or Spanish.

**Table 1:** Distribution of NENF individuals in Ontario who indicated speaking one of the top 10 non-official languages, 2006 Canadian Census

**Language**	**Total**	**% of Ontario’s total population**	**% of NENF population**
Chinese	84,415	0.7	31.81
Italian	22,900	0.19	8.63
Punjabi	21,520	0.18	8.11
Portuguese	19,360	0.16	7.3
Spanish	13,545	0.11	5.1
Urdu and/or Hindi	9,150	0.08	3.45
Vietnamese	9,135	0.08	3.44
Tamil	8,385	0.07	3.16
Arabic	6,950	0.06	2.62
Persian	6,435	0.05	2.43
Other	63,545	0.53	23.95

**Figure 2:**
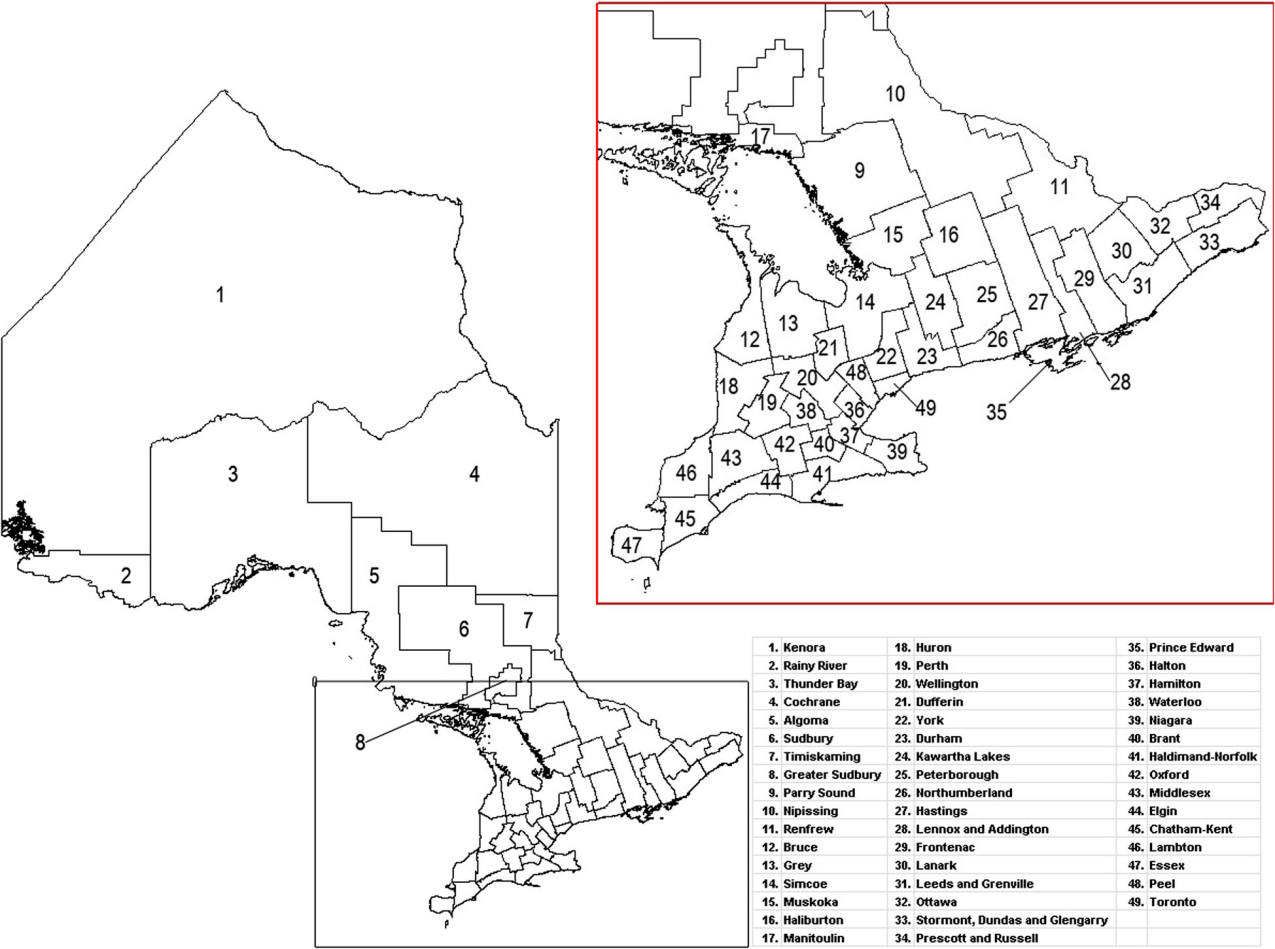
A map of Ontario broken down into 49 census divisions as defined by Statistics Canada in 2006.

**Figure 3:**
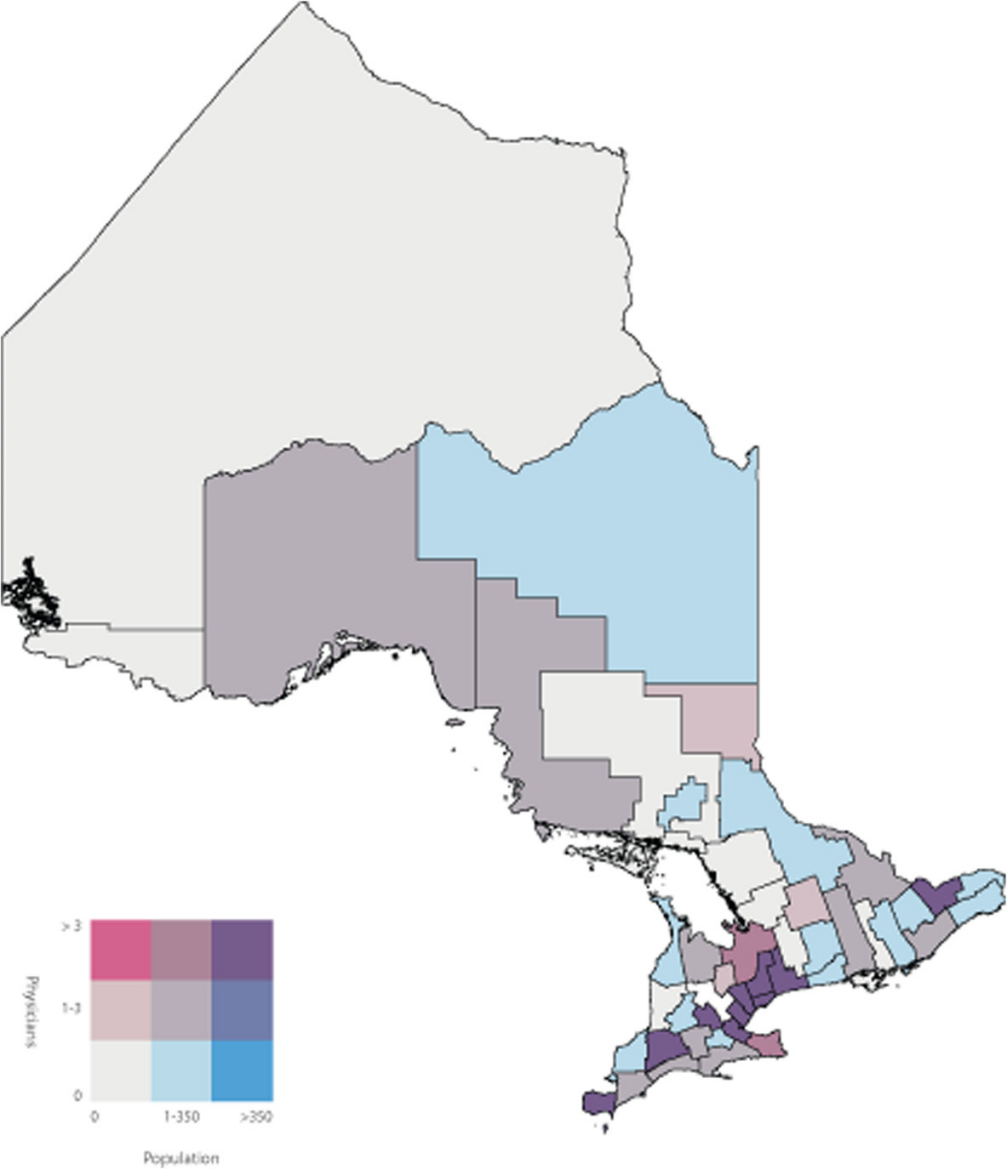
A map of the number of NENF Chinese speaking individuals and the number of primary care physicians proficient in Chinese per census division in Ontario in 2006.

**Figure 4:**
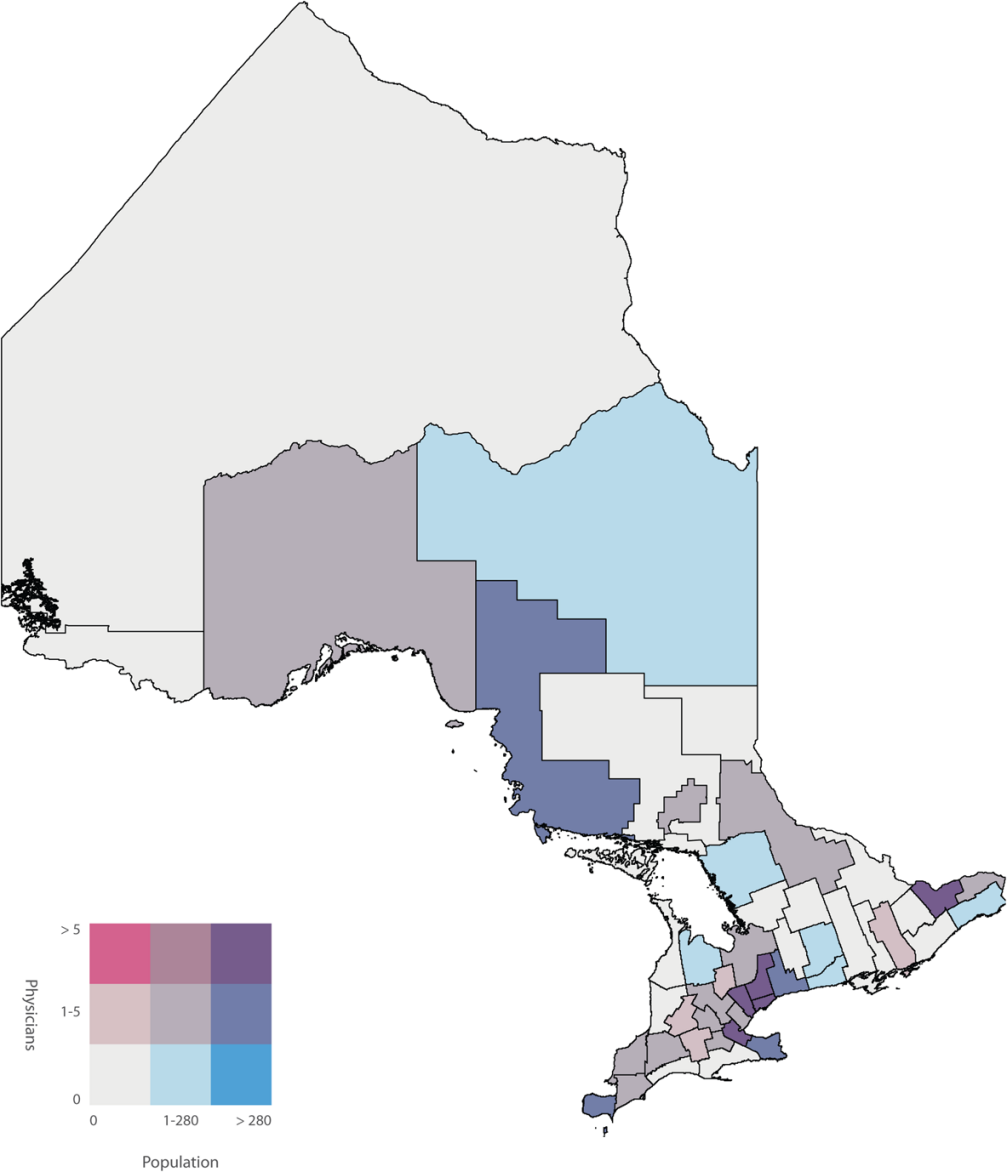
A map of the number of NENF Italian speaking individuals and the number of primary care physicians proficient in Italian per census division in Ontario in 2006.

**Figure 5:**
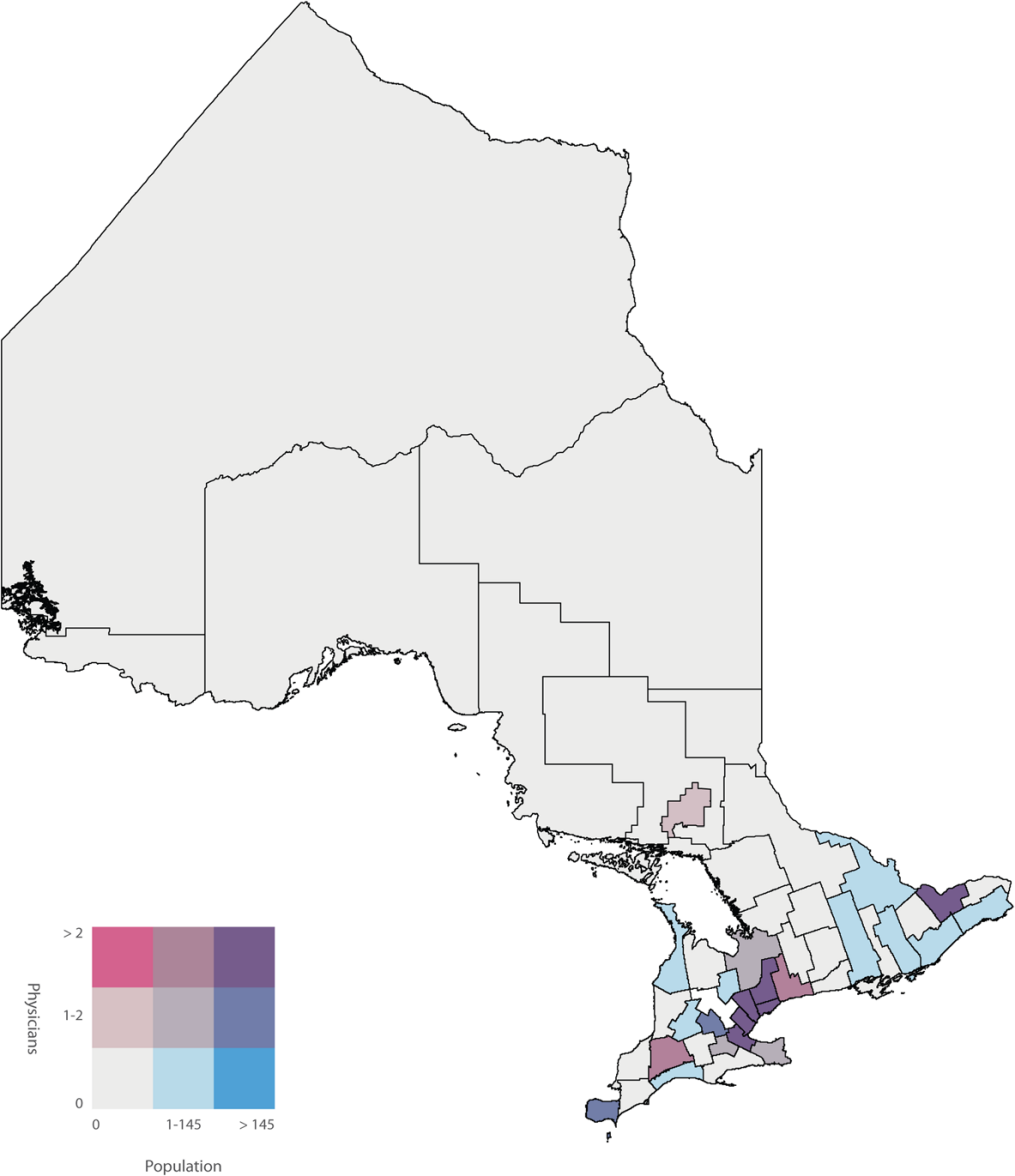
A map of the number of NENF Punjabi speaking individuals and the number of primary care physicians proficient in Punjabi per census division in Ontario in 2006.

**Figure 6:**
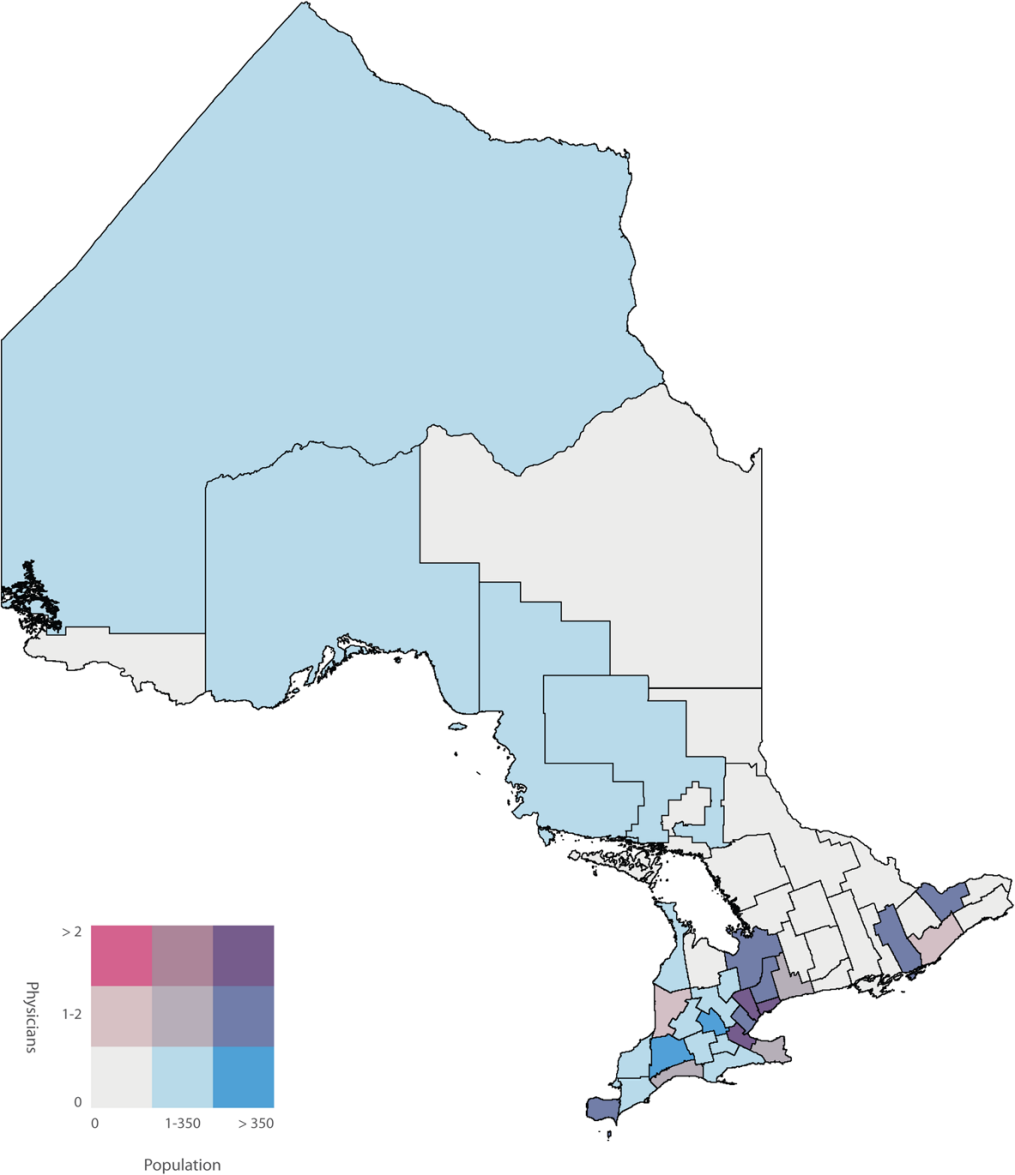
A map of the number of NENF Portuguese speaking individuals and the number of primary care physicians proficient in Portuguese per census division in Ontario in 2006.

**Figure 7:**
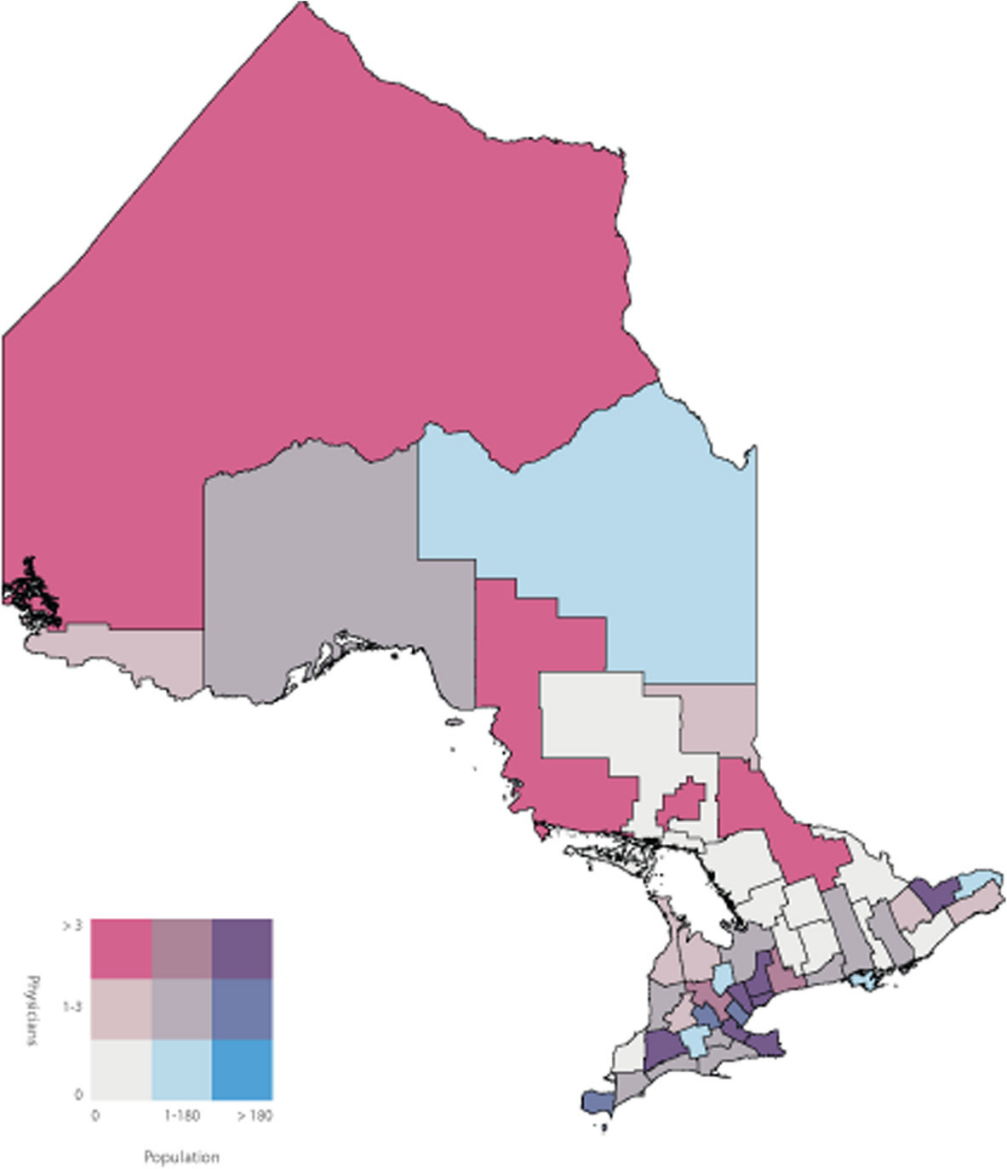
A map of the number of NENF Spanish speaking individuals and the number of primary care physicians proficient in Spanish per census division in Ontario in 2006.

A labeled map of Ontario’s census divisions is displayed in Figure 2. The spatial and linguistic mismatch of Ontario’s NENF population and Ontario’s primary care physicians proficient in the top five non-official languages are displayed in Figures 3, 4, 5, 6 and 7. There was a range of 5-15 census divisions with an NENF population speaking one of the top five non-official languages without any primary care physicians proficient in that non-official language. The size of NENF populations within these census divisions ranged from 10 individuals to 1,470 individuals. More specifically, there were 13 census divisions with a NENF Chinese speaking population in need (ranging from 5 to 350 individuals), 6 census divisions with a NENF Italian speaking population in need (ranging from 10 to 15 individuals), 9 census divisions with a NENF Punjabi speaking population in need (ranging from 10 to 145 individuals), 15 census divisions with a NENF Portuguese speaking population in need (ranging from 10 to 1,470 individuals), and 5 census divisions with a NENF Spanish speaking population in need (ranging from 10-20 individuals).

## Discussion

The purpose of this study was to quantify and visualize the linguistic and spatial mismatch between Ontario’s non-English and/or non-French (NENF) population proficient in the top five non-official languages and Ontario’s primary care physicians who are proficient in the same non-official language. The top five mother tongues spoken by the NENF populations were: Chinese, Italian, Punjabi, Portuguese and Spanish. After comparing the spatial distribution of Ontario’s NENF population and Ontario’s primary care physicians speaking the top five non-official languages, we found that, for all of the top five non-official languages, there were at least five census divisions that had a NENF population speaking that language without *any* physician capable of speaking any of the same non-official languages. However, we did find that, for Chinese, Italian, and Punjabi, the three census divisions with the largest NENF populations had the largest number of physicians proficient in that non-official language. The greatest linguistic and spatial mismatch was observed for NENF Portuguese speaking individuals wherein there were 15 census divisions with an NENF Portuguese speaking population without any physicians proficient in Portuguese. Specifically, the census division of Waterloo had 1,470 NENF Portuguese speaking individuals and no Portuguese speaking physicians.

Chinese and Punjabi languages are among the top five non-official languages spoken by Ontario’s NENF population; this is not surprising since over 60% of new immigrants arrived from Asian countries in 2006 [15]. While the number of immigrants from European countries has decreased over the last forty years [15], we did find Italian, Portuguese and Spanish to be among the top five non-official languages spoken by Ontario’s NENF population. Whether NENF Italian, Portuguese and Spanish populations are part of an earlier cohort that immigrated years ago and never learned English or French well enough to conduct a conversation is unknown, and warrants further research [15].

There have been very few studies that have looked at the spatial distribution of NENF populations and primary care physicians proficient in non-official languages. A study performed in California, USA, a state that has a large Latin American and Chinese population, found that physicians with self-reported proficiency in Spanish and Chinese languages were more likely to practice in linguistically designated neighborhoods, areas where there were patients requiring services in non-official languages, compared to physicians who only spoke English [16]. While these findings contrast with our finding that a range of 5-15 of Ontario’s census divisions had an NENF population speaking non-official languages in need of a physicians proficient in that language, we did find that, with the exception of the NENF Portuguese and Spanish populations, the top three census divisions with the largest NENF populations had highest number of physicians proficient in that non-official language. Further research would be required to determine if this finding supports the notion that physicians with proficiency in non-official languages do indeed practice in census divisions that have the largest NENF population or if it is simply a correlation. We did find that a majority of the NENF population and a majority of the physicians speaking the top five non-official languages reside and practice in the census divisions of Toronto, York or Peel. This is also in line with previous reports that stated that new immigrants as well as physicians tend to settle in larger cities [15,17].

Understanding the linguistic capabilities of Ontario’s immigrant population and the linguistic capabilities of Ontario’s primary care physicians is essential in reducing the potential risk of language discordant clinical encounters in Ontario. As immigration continues to increase, we may find that the linguistic needs of Ontario’s immigrant population diverge from the linguistic capabilities of Ontario’s primary care physicians. Potential solutions to this discordance may include interventions for both the immigrant population and the primary care physician population. One option is to increase the availability of programs for English and French language training for new immigrants. Another potential solution to this problem may be to increase the enrollment of students proficient in non-official languages into Canadian medical schools, however it would be a number of years before these students would be eligible to practice medicine. We could also consider incentives for international medical graduates, who are proficient in non-official languages, to practice in census divisions that have a NENF population in need of their linguistic skills. As part of the process in obtaining a license to practice in Ontario, international medical graduates have to practice in underserviced communities as a ‘return of service’ [18]. Currently these underserviced areas are classified based on population (count and density), travel time to a basic referral centre, and travel time to an advanced referral centre [19]. Perhaps, Ontario’s licensing bodies could allow international medical graduates to conduct their ‘return of service’ in census divisions who have a NENF population speaking a language that physician is proficient in. Not only would this be beneficial to the NENF population, but it may also be beneficial to the physicians themselves as international medical graduates may find a community of individuals with a similar culture and choose to set up their practice in that census division after their return of service is completed. That being said, it would be important to ensure that practices established by international medical graduates in these communities adhere to Ontario regulations. Finally, while previous research indicates that it is ideal for the physician and the patient to be proficient in the same language, another solution might be to increase the availability of interpretation services in the primary care setting. Previous research on the use of professional interpreters in patient-physician language discordant clinical settings has been associated with improved clinical outcomes when compared to patient-physician language discordant clinical encounters that do not use interpreters [20]. This would include providing immigrant groups and/or primary care physician practices with funding for interpretation services as well as educating physicians on the use and availability of interpretation services.

We should cautiously interpret the results of this study. One limitation to our study is that we assumed that NENF populations and primary care physicians did not cross census boundaries. There may have been an NENF population located on the border of one census division and a primary care physician located at the border of the adjacent census division, but this would not have been captured in our results. We also assumed that NENF individuals located the census divisions with physicians proficient in non-official languages are actually using those physicians, or are aware of their existence should they require medical attention, where this might not be the case. Another limitation is that linguistic capabilities in both the Canadian census and the physician database are self-reported. While both Statistics Canada and the Canadian Medical Directory go to great lengths to ensure the data are accurate, it is possible we are under reporting the linguistic capabilities of both the immigrant population and the physician population. Lastly, a major limitation our study had was that, due to the difference in the definition of Chinese languages in the Canadian census and the physician’s database, we combined all Chinese languages together. The NENF Chinese population represented 31.8% of the NENF population, but since Chinese dialects are quite different, we may have over-estimated the language concordance in certain census divisions. Despite its limitations, this is the first study to assess the spatial distribution of Ontario’s NENF population speaking the top five non-official languages and Ontario’s physicians proficient in the top five non-official languages across Ontario’s 49 census divisions.

## Conclusions

In conclusion, further research on the language discordance in Ontario is needed in order to reduce the risk of language discordant clinical encounters and the negative health outcomes associated with these encounters. Given the temporal trends in immigration in Ontario, an extension of this study would be to compare our results with those of the 2011 Canadian census and 2011 physician database. Another extension would be to further investigate the spatial and linguistic mismatch of NENF Chinese speaking individuals and Chinese speaking physicians, focusing on the specific dialects spoken by each population. It might also be beneficial to look at the spatial and linguistic mismatch on a smaller geographical scale in order to see the distance between an NENF population and proficient primary care physicians within a census division. Ideally, the distribution of the NENF population and all physicians proficient in non-official languages should be publically available (i.e. via the internet) and updated routinely. Not only would this help NENF individuals find physicians proficient in their language, it would also help new physicians identify potential locations where to set up their practice, by identifying communities in need of access to their primary language. Moreover, this information would also assist in the alignment of other interpretation and related social services, and individuals whose language barriers are preventing them from accessing health care services may also be preventing them from accessing essential social services as well.

## Competing interests

The authors declare that they have no competing interests.

## Author’s contributions

JS participated in the design and coordination of the study, performed the statistical and spatial analysis required for the study and drafted the manuscript. KK assisted in the conception and design of the study, provided data from the Canadian Medical Directory Physician’s file and helped to draft the manuscript. CA participated in the coordination of the study and helped to draft the manuscript. HT participated in the design and coordination of the study, supervised the statistical and spatial analysis and helped to draft the manuscript. All authors read and approved the final manuscript.

## Pre-publication history

The pre-publication history for this paper can be accessed here:

http://www.biomedcentral.com/1472-6963/13/535/prepub
